# Supplementary material for: Causal Relationship between Aging and Anorexia Nervosa: A White-Matter-Microstructure-Mediated Mendelian Randomization Analysis
Source: Biomedicines. 2024 Aug 16;12(8):1874. doi: 10.3390/biomedicines12081874 (PMC11351342; doi:10.3390/biomedicines12081874)
Supplement: Supplementary file 1 [file biomedicines-12-01874-s001.zip › Supplementary Figure/Figure S4/Singe SNP plot of MR analysis for TL to ubm-b-1514.pdf]

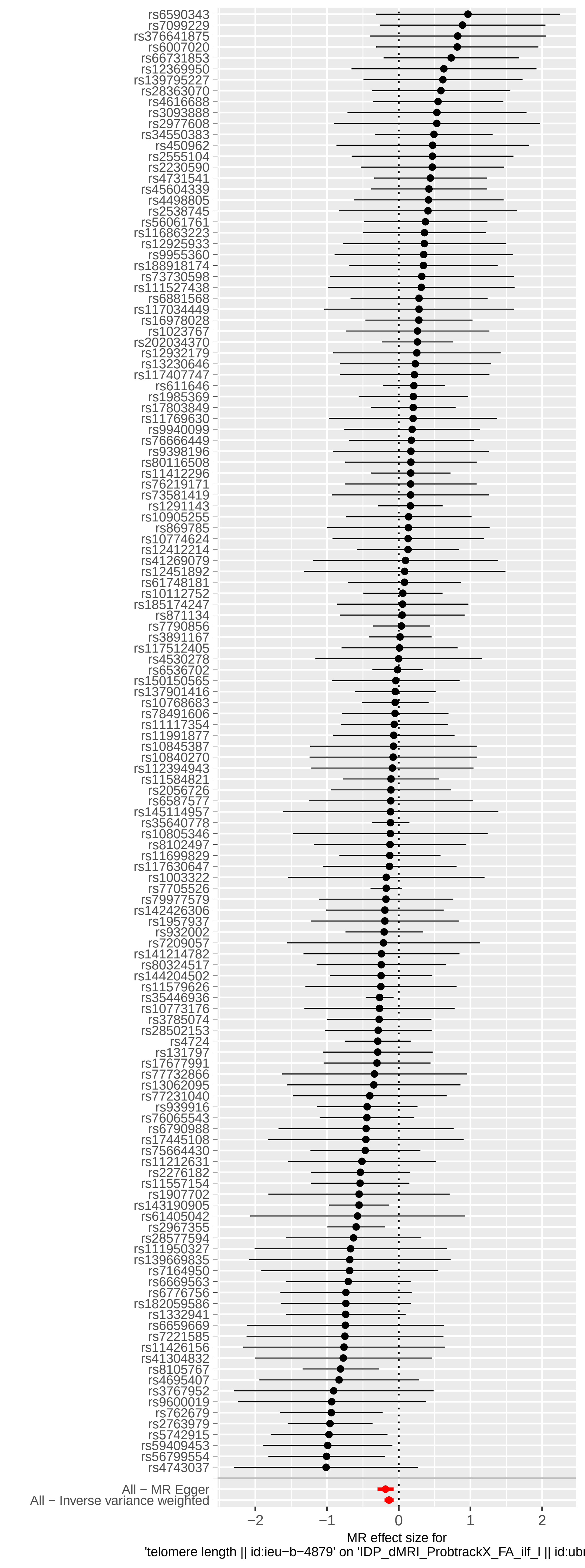

rs6590343
rs7099229
rs376641875
rs6007020
rs66731853
rs12369950
rs139795227
rs28363070
rs4616688
rs3093888
rs2977608
rs34550383
rs450962
rs2555104
rs2230590
rs4731541
rs45604339
rs4498805
rs2538745
rs56061761
rs116863223
rs12925933
rs9955360
rs188918174
rs73730598
rs111527438
rs6881568
rs117034449
rs16978028
rs1023767
rs202034370
rs12932179
rs13230646
rs117407747
rs611646
rs1985369
rs17803849
rs11769630
rs9940099
rs76666449
rs9398196
rs80116508
rs11412296
rs76219171
rs73581419
rs1291143
rs10905255
rs869785
rs10774624
rs12412214
rs41269079
rs12451892
rs61748181
rs10112752
rs185174247
rs871134
rs7790856
rs3891167
rs117512405
rs4530278
rs6536702
rs150150565
rs137901416
rs10768683
rs78491606
rs11117354
rs11991877
rs10845387
rs10840270
rs112394943
rs11584821
rs2056726
rs6587577
rs145114957
rs35640778
rs10805346
rs8102497
rs11699829
rs117630647
rs1003322
rs7705526
rs79977579
rs142426306
rs1957937
rs932002
rs7209057
rs141214782
rs80324517
rs144204502
rs11579626
rs35446936
rs10773176
rs3785074
rs28502153
rs4724
rs131797
rs17677991
rs77732866
rs13062095
rs77231040
rs939916
rs76065543
rs6790988
rs17445108
rs75664430
rs11212631
rs2276182
rs11557154
rs1907702
rs143190905
rs61405042
rs2967355
rs28577594
rs111950327
rs139669835
rs7164950
rs6669563
rs6776756
rs182059586
rs1332941
rs6659669
rs7221585
rs11426156
rs41304832
rs8105767
rs4695407
rs3767952
rs9600019
rs762679
rs2763979
rs5742915
rs59409453
rs56799554
rs4743037
All − MR Egger
All − Inverse variance weighted
−2
−1
0
1
2
MR effect size for
'telomere length || id:ieu-b-4879' on 'IDP_dMRI_ProbtrackX_FA_ilf_l || id:ubi
